# 1-[4-(1*H*-imidazol-1-yl)phen­yl]ethanone monohydrate

**DOI:** 10.1107/S1600536812029157

**Published:** 2012-06-30

**Authors:** Halliru Ibrahim, Muhammad D. Bala, Bernard Omondi

**Affiliations:** a School of Chemistry & Physics, University of KwaZulu-Natal, Westville Campus, Private Bag X54001, Durban 4000, South Africa

## Abstract

In the crystal structure of the title compound, C_11_H_10_N_2_O·H_2_O, the solvent water mol­ecule links the organic mol­ecules through O—H⋯O and O—H⋯N hydrogen bonds, forming chains that run diagonally across the *bc* face. These chains are connected to adjacent chains through weak C—H⋯O inter­actions, resulting in hydrogen-bonded sheets extending along the *b* and *c* axes. The sheets are connected along the *a* axis through π–π inter­actions, with centroid–centroid distances of 3.7571 (9) and 3.7231 (9) Å.

## Related literature

For the synthesis of the title compound, see: Corberán & Peris (2008 ▶). For the structure of imidazole analogues with bonds to the phenyl group *via* carbon, see: Gayathri *et al.* (2010 ▶). For the structure of imidazole analogues *N*-bonded to a phenyl group, see: Zheng *et al.* (2011 ▶). For structures of other related compounds, see: Ishihara *et al.* (1992 ▶).
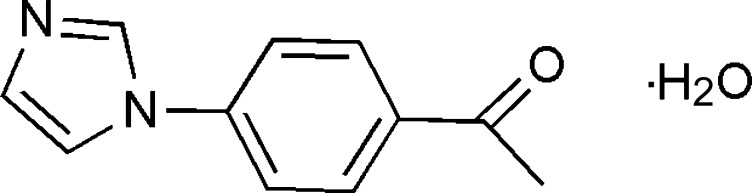


## Experimental

### 

#### Crystal data

C_11_H_10_N_2_O·H_2_O
*M* *_r_* = 204.23
Triclinic, 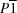

*a* = 6.7599 (6) Å
*b* = 8.0885 (8) Å
*c* = 9.7168 (9) Å
α = 90.350 (3)°
β = 106.731 (3)°
γ = 99.486 (3)°
*V* = 501.03 (8) Å^3^
*Z* = 2
Mo *K*α radiation
μ = 0.10 mm^−1^
*T* = 173 K
0.58 × 0.39 × 0.14 mm

#### Data collection

Bruker SMART APEXII CCD diffractometer
Absorption correction: multi-scan (*SADABS*; Bruker, 2008 ▶) *T*_min_ = 0.947, *T*_max_ = 0.987
6609 measured reflections
1711 independent reflections
1568 reflections with *I* > 2σ(*I*)
*R*_int_ = 0.025

#### Refinement

*R*[*F* ^2^ > 2σ(*F* ^2^)] = 0.036
*wR*(*F* ^2^) = 0.098
*S* = 1.07
1711 reflections
153 parameters
H atoms treated by a mixture of independent and constrained refinement
Δρ_max_ = 0.15 e Å^−3^
Δρ_min_ = −0.34 e Å^−3^

### 

Data collection: *APEX2* (Bruker, 2008 ▶); cell refinement: *SAINT-Plus* (Bruker, 2008 ▶); data reduction: *SAINT-Plus* and *XPREP* (Bruker, 2008 ▶); program(s) used to solve structure: *SHELXS97* (Sheldrick, 2008 ▶); program(s) used to refine structure: *SHELXL97* (Sheldrick, 2008 ▶); molecular graphics: *ORTEP-3* (Farrugia, 1997 ▶); software used to prepare material for publication: *WinGX* (Farrugia, 1999 ▶).

## Supplementary Material

Crystal structure: contains datablock(s) global, I. DOI: 10.1107/S1600536812029157/fj2569sup1.cif

Structure factors: contains datablock(s) I. DOI: 10.1107/S1600536812029157/fj2569Isup2.hkl

Supplementary material file. DOI: 10.1107/S1600536812029157/fj2569Isup3.cml

Additional supplementary materials:  crystallographic information; 3D view; checkCIF report

## Figures and Tables

**Table 1:** Hydrogen-bond geometry (Å, °)

*D*—H⋯*A*	*D*—H	H⋯*A*	*D*⋯*A*	*D*—H⋯*A*
O1*S*—H1*D*⋯O1^i^	0.87 (2)	2.00 (2)	2.8610 (14)	174.6 (19)
O1*S*—H1*E*⋯N2	0.91 (2)	1.92 (2)	2.8246 (15)	172.1 (16)
C5—H5⋯O1*S* ^ii^	0.93	2.39	3.3034 (16)	166
C9—H9⋯O1*S* ^ii^	0.948 (15)	2.345 (15)	3.2677 (16)	164.5 (12)

Symmetry codes: (i) 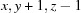; (ii) 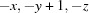.

**Fig. 1.**
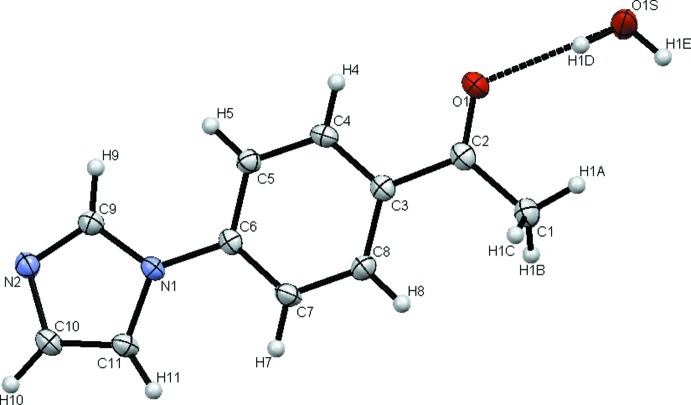
ORTEP diagram of compound (I). Thermal ellipsoids are represented at the 50% probability level.

**Fig. 2.**
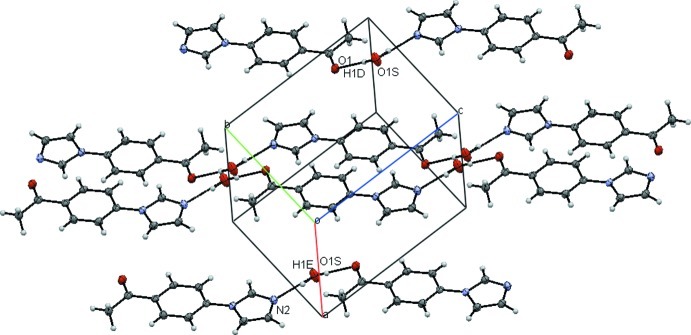
Packing diagram showing the O—H···O and N—H···O hydrogen bonds.

## supplementary crystallographic information

### Comment

The title compound is an intermediate product in the synthetic route to an *N*-heterocyclic carbene (NHC) chelating ligand bearing a pyridine backbone. The anhydrous form of the compound is available in chemical book database with CAS No. 10041–06-2. Neither structure of the hydrated nor the anhydrous forms of the title compound have been reported. Our synthetic route and the synthons used are different from those reported in the synthesis of the anhydrous form of the title compound. Absolute configuration of the title compound obtained in pure form from column chromatography using hexane:chloroform (6:4) solvent system was assigned by NMR and IR spectroscopy. Monohydrate block crystals of (I) were recrystallized from the same solvent system. The imidazole N(2) – phenyl carbon bond [C(9)—N(2)] is 1.3107 (16) Å. The one molecule of water binds as water of crystallization to the organic molecule, and is a constituent of the asymmetric unit cell. Molecules of (I) are stabilized through an extensive chain of hydrogen bonded network involving neighbouring methoxy (O—H···O) and imidazolium (N—H···O) moieties linked by the water of crystallization. Imidazole analogues of (I) with bonds to the phenyl group *via* carbon have been reported by Gayathri *et al.* (2010); while Zheng *et al.* (2011) have reported imidazole bonded to a phenyl group *via* nitrogen. Other related compounds have been reported by Ishihara *et al.* (1992).

### Experimental

The compound was synthesized by the modification of the method of Corberán *et al.* (2008). A 150 ml round bottom flask containing imidazole (0.01 mol, 0.68 g, Fluka AG) with KOH (0.015 mol, 0.84 g, Merck) was stirred at room temperature in DMSO (30 ml, Merck) for 2 h. Thereafter, *para-*choloroacetophenone (0.01 mol, 1.34 ml, Aldrich) was added dropwise, and then refluxed at 100 °C for 24 h. The reaction mixture was then allowed to cool down to room temperature, washed and diluted with chilled distilled water till it became neutral. Addition of distilled water to the contents of the reaction flask gave a muddy emulsion which took 24 h to partition when extracted with chloroform (6x10 ml). The resulting organic components were dried in anhydrous MgSO_4_ and concentrated *in vacuo* yielding crude dark brown oily liquid (1.694 g). Thin layer chromatography of the crude product showed that it contained the expected product (*R*_f_ value 0.45 in ethyl acetate:methanol 4:6 solvent system) contaminated with unreacted imidazole and *para-*chloroacetophenone. Column chromatography of the crude product using hexane:chlorofom solvent system afforded the title compound as block-shaped light green crystals (0.987 g, 48.4% yield), m.p. = 119–122 °C. ^1^H NMR (400 MHz, CDCl_3_): 8.09(*d*), 7.95(*s*), 7.51(*d*), 7.35(*s*), 7.25(*s*), and 1.62(*s*). ^13^C NMR (400 MHz, CDCl_3_): 196.5 (carbonyl CO), 140.8, 135.8, 135.4, 131.2, 130.4, 120.7, 117.7, 26.61(CH_3_). IR (ATR, cm^-1^): 1665(C=O), 1606(C=N), 1530(N—C), 956(C=C), 2222(C—H), 814(*para* substituted benzene), 3202 (water of crystallization absorbance band).

### Refinement

Carbon-bound H-atoms were placed in calculated positions [C—H = 0.96 Å for Me H atoms and 0.93 Å for aromatic H atoms; *U*_iso_(H) = 1.2*U*_eq_(C) (1.5 for Me groups)] and were included in the refinement in the riding model approximation. The O—H H-atom was located in a difference map and freely refined with O—H = 0.87 – 0.91 Å (*U*_iso_(H) = 1.2*U*_eq_(O).

### Crystal data

**Table:**

C_11_H_10_N_2_O·H_2_O	*Z* = 2
*M**_r_* = 204.23	*F*(000) = 216
Triclinic, *P*1	*D*_x_ = 1.354 Mg m^−^^3^
Hall symbol: -P 1	Mo *K*α radiation, λ = 0.71073 Å
*a* = 6.7599 (6) Å	Cell parameters from 7553 reflections
*b* = 8.0885 (8) Å	θ = 2.2–25.0°
*c* = 9.7168 (9) Å	µ = 0.10 mm^−^^1^
α = 90.350 (3)°	*T* = 173 K
β = 106.731 (3)°	Block, colourless
γ = 99.486 (3)°	0.58 × 0.39 × 0.14 mm
*V* = 501.03 (8) Å^3^	

### Data collection

**Table:**

Bruker SMART APEXII CCD diffractometer	1568 reflections with *I* > 2σ(*I*)
Graphite monochromator	*R*_int_ = 0.025
φ and ω scans	θ_max_ = 25.0°, θ_min_ = 2.2°
Absorption correction: multi-scan (*SADABS*; Bruker, 2008)	*h* = −7→8
*T*_min_ = 0.947, *T*_max_ = 0.987	*k* = −9→9
6609 measured reflections	*l* = −11→11
1711 independent reflections	

### Refinement

**Table:**

Refinement on *F*^2^	Primary atom site location: structure-invariant direct methods
Least-squares matrix: full	Secondary atom site location: difference Fourier map
*R*[*F*^2^ > 2σ(*F*^2^)] = 0.036	Hydrogen site location: inferred from neighbouring sites
*wR*(*F*^2^) = 0.098	H atoms treated by a mixture of independent and constrained refinement
*S* = 1.07	*w* = 1/[σ^2^(*F*_o_^2^) + (0.052*P*)^2^ + 0.1639*P*] where *P* = (*F*_o_^2^ + 2*F*_c_^2^)/3
1711 reflections	(Δ/σ)_max_ = 0.002
153 parameters	Δρ_max_ = 0.15 e Å^−^^3^
0 restraints	Δρ_min_ = −0.34 e Å^−^^3^

### Special details

**Table:**

Experimental. Carbon-bound H-atoms were placed in calculated positions [C—H = 0.96 Å for Me H atoms and 0.93 Å for aromatic H atoms; *U*_iso_(H) = 1.2*U*_eq_(C) (1.5 for Me groups)] and were included in the refinement in the riding model approximation. The O—H H-atom was located in a difference map and freely refined with O—H = 0.87 – 0.91 Å (*U*_iso_(H) = 1.2*U*_eq_(O).
Geometry. All e.s.d.'s (except the e.s.d. in the dihedral angle between two l.s. planes) are estimated using the full covariance matrix. The cell e.s.d.'s are taken into account individually in the estimation of e.s.d.'s in distances, angles and torsion angles; correlations between e.s.d.'s in cell parameters are only used when they are defined by crystal symmetry. An approximate (isotropic) treatment of cell e.s.d.'s is used for estimating e.s.d.'s involving l.s. planes. The following ALERTS were generated. Each ALERT has the format test-name_ALERT_alert-type_alert-level. Alert level C PLAT029_ALERT_3_C _diffrn_measured_fraction_theta_full Low ······. 0.971 PLAT911_ALERT_3_C Missing # FCF Refl Between THmin & STh/*L*= 0.594 51 PLAT154_ALERT_1_G The su's on the Cell Angles are Equal ······.0.00300 Deg. PLAT764_ALERT_4_G Overcomplete CIF Bond List Detected (Rep/Expd) 1.20 Ratio PLAT790_ALERT_4_G Centre of Gravity not Within Unit Cell: Resd. #2 H2 O PLAT909_ALERT_3_G Percentage of Observed Data at Theta(Max) still 84 Perc. NOTED:
Refinement. Refinement of *F*^2^ against ALL reflections. The weighted *R*-factor *wR* and goodness of fit *S* are based on *F*^2^, conventional *R*-factors *R* are based on *F*, with *F* set to zero for negative *F*^2^. The threshold expression of *F*^2^ > σ(*F*^2^) is used only for calculating *R*-factors(gt) *etc*. and is not relevant to the choice of reflections for refinement. *R*-factors based on *F*^2^ are statistically about twice as large as those based on *F*, and *R*- factors based on ALL data will be even larger.

### Fractional atomic coordinates and isotropic or equivalent isotropic displacement parameters (Å^2^)

**Table:**

	*x*	*y*	*z*	*U*_iso_*/*U*_eq_	
C1	0.3585 (2)	0.18679 (16)	0.96056 (14)	0.0245 (3)	
H1A	0.3397	0.0859	1.0103	0.037*	
H1B	0.2732	0.2621	0.9808	0.037*	
H1C	0.5033	0.2395	0.9923	0.037*	
C2	0.29495 (19)	0.14436 (15)	0.80160 (14)	0.0195 (3)	
C3	0.28326 (18)	0.28277 (15)	0.70069 (13)	0.0174 (3)	
C4	0.21354 (19)	0.24258 (15)	0.55292 (14)	0.0184 (3)	
H4	0.1746	0.1304	0.5199	0.022*	
C5	0.20107 (19)	0.36543 (15)	0.45475 (13)	0.0182 (3)	
H5	0.1554	0.3361	0.3566	0.022*	
C6	0.25730 (17)	0.53395 (14)	0.50336 (13)	0.0160 (3)	
C7	0.32455 (19)	0.57689 (15)	0.65011 (14)	0.0204 (3)	
H7	0.3611	0.6892	0.6829	0.024*	
C8	0.3369 (2)	0.45188 (16)	0.74724 (14)	0.0208 (3)	
H8	0.3818	0.4813	0.8454	0.025*	
C9	0.17884 (19)	0.63657 (15)	0.25569 (14)	0.0195 (3)	
C10	0.24881 (19)	0.90217 (15)	0.30220 (14)	0.0206 (3)	
C11	0.28704 (19)	0.83293 (15)	0.43105 (14)	0.0197 (3)	
H11	0.3339	0.8894	0.5213	0.024*	
N1	0.24241 (15)	0.66069 (12)	0.40191 (11)	0.0165 (3)	
N2	0.18116 (16)	0.77885 (13)	0.19181 (11)	0.0210 (3)	
O1	0.25470 (14)	−0.00186 (10)	0.75446 (10)	0.0248 (3)	
O1S	0.04729 (17)	0.73832 (13)	−0.11171 (11)	0.0353 (3)	
H1D	0.115 (3)	0.820 (3)	−0.147 (2)	0.053 (5)*	
H1E	0.093 (3)	0.762 (2)	−0.015 (2)	0.047 (5)*	
H9	0.137 (2)	0.5281 (19)	0.2087 (16)	0.024 (4)*	
H10	0.262 (2)	1.0191 (17)	0.2805 (14)	0.017 (3)*	

### Atomic displacement parameters (Å^2^)

**Table:**

	*U*^11^	*U*^22^	*U*^33^	*U*^12^	*U*^13^	*U*^23^
C1	0.0292 (7)	0.0211 (6)	0.0227 (7)	0.0052 (5)	0.0064 (6)	0.0051 (5)
C2	0.0160 (6)	0.0193 (6)	0.0237 (7)	0.0030 (5)	0.0067 (5)	0.0031 (5)
C3	0.0146 (6)	0.0177 (6)	0.0208 (7)	0.0032 (5)	0.0061 (5)	0.0020 (5)
C4	0.0180 (6)	0.0135 (6)	0.0233 (7)	0.0021 (5)	0.0058 (5)	−0.0010 (5)
C5	0.0186 (6)	0.0180 (6)	0.0169 (6)	0.0027 (5)	0.0039 (5)	−0.0004 (5)
C6	0.0122 (6)	0.0169 (6)	0.0193 (7)	0.0025 (5)	0.0054 (5)	0.0029 (5)
C7	0.0229 (6)	0.0132 (6)	0.0233 (7)	0.0010 (5)	0.0053 (5)	−0.0007 (5)
C8	0.0233 (7)	0.0206 (6)	0.0163 (6)	0.0020 (5)	0.0036 (5)	−0.0005 (5)
C9	0.0208 (7)	0.0177 (6)	0.0193 (7)	0.0014 (5)	0.0057 (5)	0.0006 (5)
C10	0.0217 (6)	0.0152 (6)	0.0246 (7)	0.0020 (5)	0.0069 (5)	0.0032 (5)
C11	0.0202 (6)	0.0145 (6)	0.0224 (7)	0.0007 (5)	0.0043 (5)	−0.0010 (5)
N1	0.0161 (5)	0.0138 (5)	0.0187 (6)	0.0014 (4)	0.0047 (4)	0.0015 (4)
N2	0.0228 (6)	0.0189 (5)	0.0207 (6)	0.0016 (4)	0.0063 (4)	0.0031 (4)
O1	0.0330 (5)	0.0163 (5)	0.0251 (5)	0.0027 (4)	0.0094 (4)	0.0030 (4)
O1S	0.0489 (7)	0.0281 (5)	0.0209 (6)	−0.0152 (5)	0.0102 (5)	−0.0023 (4)

### Geometric parameters (Å, º)

**Table:**

C1—C2	1.5003 (18)	C7—H7	0.93
C1—H1A	0.96	C8—H8	0.93
C1—H1B	0.96	C9—N2	1.3109 (16)
C1—H1C	0.96	C9—N2	1.3109 (16)
C2—O1	1.2240 (15)	C9—N1	1.3634 (17)
C2—C3	1.4896 (17)	C9—H9	0.948 (15)
C3—C8	1.3935 (17)	C10—C11	1.3483 (19)
C3—C4	1.3941 (18)	C10—N2	1.3816 (17)
C4—C5	1.3775 (17)	C10—N2	1.3816 (17)
C4—H4	0.93	C10—H10	0.966 (14)
C5—C6	1.3941 (17)	C11—N1	1.3855 (15)
C5—H5	0.93	C11—H11	0.93
C6—C7	1.3890 (18)	O1S—H1D	0.87 (2)
C6—N1	1.4220 (15)	O1S—H1E	0.91 (2)
C7—C8	1.3835 (18)		
			
C2—C1—H1A	109.5	C6—C7—H7	120.1
C2—C1—H1B	109.5	C7—C8—C3	121.20 (12)
H1A—C1—H1B	109.5	C7—C8—H8	119.4
C2—C1—H1C	109.5	C3—C8—H8	119.4
H1A—C1—H1C	109.5	N2—C9—N1	112.07 (11)
H1B—C1—H1C	109.5	N2—C9—N1	112.07 (11)
O1—C2—C3	119.93 (11)	N2—C9—H9	125.6 (9)
O1—C2—C1	120.84 (11)	N2—C9—H9	125.6 (9)
C3—C2—C1	119.23 (11)	N1—C9—H9	122.3 (9)
C8—C3—C4	118.11 (11)	C11—C10—N2	110.55 (11)
C8—C3—C2	122.91 (11)	C11—C10—N2	110.55 (11)
C4—C3—C2	118.98 (11)	C11—C10—H10	129.4 (8)
C5—C4—C3	121.44 (11)	N2—C10—H10	120.0 (8)
C5—C4—H4	119.3	N2—C10—H10	120.0 (8)
C3—C4—H4	119.3	C10—C11—N1	106.12 (11)
C4—C5—C6	119.63 (11)	C10—C11—H11	126.9
C4—C5—H5	120.2	N1—C11—H11	126.9
C6—C5—H5	120.2	C9—N1—C11	106.12 (10)
C7—C6—C5	119.90 (11)	C9—N1—C6	126.67 (10)
C7—C6—N1	120.52 (11)	C11—N1—C6	127.20 (10)
C5—C6—N1	119.58 (11)	C9—N2—C10	105.14 (10)
C8—C7—C6	119.72 (11)	H1D—O1S—H1E	104.5 (17)
C8—C7—H7	120.1		
			
O1—C2—C3—C8	−176.76 (11)	N2—C9—N1—C11	0.29 (14)
C1—C2—C3—C8	2.59 (18)	N2—C9—N1—C11	0.29 (14)
O1—C2—C3—C4	3.98 (18)	N2—C9—N1—C6	179.67 (10)
C1—C2—C3—C4	−176.67 (11)	N2—C9—N1—C6	179.67 (10)
C8—C3—C4—C5	1.18 (18)	C10—C11—N1—C9	−0.15 (13)
C2—C3—C4—C5	−179.52 (10)	C10—C11—N1—C6	−179.53 (11)
C3—C4—C5—C6	−0.60 (18)	C7—C6—N1—C9	−179.88 (11)
C4—C5—C6—C7	−0.27 (18)	C5—C6—N1—C9	−0.77 (18)
C4—C5—C6—N1	−179.39 (10)	C7—C6—N1—C11	−0.63 (18)
C5—C6—C7—C8	0.51 (18)	C5—C6—N1—C11	178.48 (11)
N1—C6—C7—C8	179.62 (11)	N1—C9—N2—N2	0.0 (3)
C6—C7—C8—C3	0.10 (19)	N2—C9—N2—C10	0E1 (10)
C4—C3—C8—C7	−0.93 (19)	N1—C9—N2—C10	−0.30 (14)
C2—C3—C8—C7	179.80 (11)	C11—C10—N2—N2	0.00 (19)
N2—C10—C11—N1	−0.02 (14)	C11—C10—N2—C9	0.20 (14)
N2—C10—C11—N1	−0.02 (14)	N2—C10—N2—C9	0E1 (8)

### Hydrogen-bond geometry (Å, º)

**Table:**

*D*—H···*A*	*D*—H	H···*A*	*D*···*A*	*D*—H···*A*
O1*S*—H1*D*···O1^i^	0.87 (2)	2.00 (2)	2.8610 (14)	174.6 (19)
O1*S*—H1*E*···N2	0.91 (2)	1.92 (2)	2.8246 (15)	172.1 (16)
C5—H5···O1*S*^ii^	0.93	2.39	3.3034 (16)	166
C9—H9···O1*S*^ii^	0.948 (15)	2.345 (15)	3.2677 (16)	164.5 (12)

Symmetry codes: (i) *x*, *y*+1, *z*−1; (ii) −*x*, −*y*+1, −*z*.
